# Development of a Sequence Searchable Database of Celiac Disease-Associated Peptides and Proteins for Risk Assessment of Novel Food Proteins

**DOI:** 10.3389/falgy.2022.900573

**Published:** 2022-05-26

**Authors:** Plaimein Amnuaycheewa, Mohamed Abdelmoteleb, John Wise, Barbara Bohle, Fatima Ferreira, Afua O. Tetteh, Steve L. Taylor, Richard E. Goodman

**Affiliations:** ^1^Department of Agro-Industrial, Food, and Environmental Technology, King Mongkut's University of Technology North Bangkok (KMUTNB), Bangkok, Thailand; ^2^Botany Department, Faculty of Science, Mansoura University, Mansoura, Egypt; ^3^Food Allergy Research and Resource Program (FARRP), Department of Food Science and Technology, University of Nebraska, Lincoln, NE, United States; ^4^Christian Doppler Laboratory for Immunomodulation, Department of Pathophysiology and Allergy Research, Medical University of Vienna, Vienna, Austria; ^5^Department of Biosciences, University of Salzburg, Salzburg, Austria; ^6^BASF Corporation, Morrisville, NC, United States

**Keywords:** celiac disease, gluten, T-cell epitopes, peptide database, risk assessment, sequence comparison, Pooideae, prolamin

## Abstract

Celiac disease (CeD) is an autoimmune enteropathy induced by prolamin and glutelin proteins in wheat, barley, rye, and triticale recognized by genetically restricted major histocompatibility (MHC) receptors. Patients with CeD must avoid consuming these proteins. Regulators in Europe and the United States expect an evaluation of CeD risks from proteins in genetically modified (GM) crops or novel foods for wheat-related proteins. Our database includes evidence-based causative peptides and proteins and two amino acid sequence comparison tools for CeD risk assessment. Sequence entries are based on the review of published studies of specific gluten-reactive T cell activation or intestinal epithelial toxicity. The initial database in 2012 was updated in 2018 and 2022. The current database holds 1,041 causative peptides and 76 representative proteins. The FASTA sequence comparison of 76 representative CeD proteins provides an insurance for possible unreported epitopes. Validation was conducted using protein homologs from Pooideae and non-Pooideae monocots, dicots, and non-plant proteins. Criteria for minimum percent identity and maximum *E*-scores are guidelines. Exact matches to any of the 1,041 peptides suggest risks, while FASTA alignment to the 76 CeD proteins suggests possible risks. Matched proteins should be tested further by CeD-specific CD4/8+ T cell assays or *in vivo* challenges before their use in foods.

## Introduction

Novel proteins and complex foods are being introduced into the human diet through the creation of genetically engineered organisms, by the addition of isolated proteins, and by the introduction of novel organisms with limited or no history of safe human consumption (1). Prior to marketing, novel proteins and foods containing proteins from species related to wheat should undergo a safety evaluation to ensure safe consumption by those with CeD. The 2003 Codex Alimentarius Commission guideline called for evaluating proteins encoded by genes transferred from wheat and wheat relatives into a different species to be evaluated for potential risks of eliciting CeD as part of the overall food safety evaluation (2).

Celiac disease involves gluten-mediated inflammation that damages the epithelial lining of the upper small intestine leading to villous flattening and nutrient malabsorption. Extra-intestinal manifestations of the chronic enteropathy include short stature, osteopenia, dermatitis herpetiformis, and ataxia (3–5). Life-long, strict adherence to a gluten-free diet reverses the damage and function. The causative gluten proteins are the seed storage prolamins and glutelins, which in wheat are called gliadins and glutenins, respectively. The prolamins are called hordeins in barley and secalins in rye. Among plant-derived, dietary proteins, prolamins and glutelins contain a high amount of proline (P) and glutamine (Q) amino acids in their sequences contributing to the visco-elastic property of bread dough and conferring resistance to gastrointestinal digestion (6). Many digestion-resistant gluten peptides are found to travel across intestinal epithelium via transcellular absorption or through stimulating CXC chemokine receptor 3 (7, 8). The peptides then bind to human leukocyte antigen (HLA) DQ2.5 (DRB1^*^301-DQA1^*^0501-DQB1^*^0201) or DQ8 (DRB1^*^04-DQA1^*^0301-DQB1^*^0302) on antigen-presenting cells and are presented to activate pro-inflammatory T cells (9, 10). In Europe and the United Kingdom, more than 90% of patients with CeD express HLA-DQ2.5 and 5–10% of the patients express HLA-DQ8 (11–13). The percentage of patients with CeD patients in the United States carrying the genotypes has been estimated to be 82 and 16%, respectively (14). A multicenter study reported that nearly 0.4% of the patients carry DR5-DQ7 (DRB1^*^11/12-DQA1^*^0505-DQB1^*^0301) or DR7-DQ2 (DRB1^*^07-DQA1^*^0201-DQB1^*^0202), which can form a heterozygous DQ2.5 (DQA1^*^0505-DQB1^*^0202) binding groove (15). These MHC receptors present both native and tissue transglutaminase (TG2) deamidated peptides to the reactive cluster of differentiation 4 (CD4) T cells, and the TG2-gluten peptide complex can be a target of the activated T cells (16). Studies demonstrated that DQ2.5 preferentially binds to peptides having a 9-mer binding core with negatively charged anchors at positions 4 and 6 or 7, whereas the DQ8 allele preferentially binds peptides with negatively charged anchors at positions 1 and 9. To a lesser extent, DQ2.5 and DQ8 alleles preferentially bind to peptides with P at positions 1 and 6, respectively (17–22). Digestion-resistant gluten peptides lack polar acidic amino acids, yet the position of specific amino acids in these peptides allows TG2 deamidation at appropriately spaced Q residues, converting them to glutamic acid (E). Deamidation is important for the selection of T-cell epitopes since most of the DQ2.5 recognized epitopes are in the deamidated form (17, 20, 23–26). For example, a number of deamidated positions were reported on the 33-mer, CD4+ T cell reactive alpha2-gliadin peptide (LQLQPFPQPQLPYPQPQLPYPQPQLPYPQPQPF) (27). The DQ8 molecule recognizes the epitopes equally in both native and deamidated forms (28). The specificity of TG2 deamidation of the peptides has not been conclusively demonstrated, but some residues are more effectively modified in peptides with the configuration of QXP, where X represents amino acids other than P (25, 27, 29). The MHC restriction is predictive, but not definitive, since nearly 40% of the general population carries HLA-DQ2 or HLA-DQ8 genes, but only 1% of the population exhibit CeD (30). Genome-wide association studies indicated that non-HLA, immune-related genes also relate to CeD (31–33).

The adaptive immune response to gluten proteins appears to involve both helper and cytotoxic T cells. An α-gliadin peptide (p123-p132: QLIPCMDVVL) induced HLA-A2 specific CD8+ T cells isolated from biopsies of DQ2/DQ8 CeD patients to undergo maturation to express Fas ligand and secrete interferon-gamma (IFN-γ) and granzyme B (34). The CeD pathogenesis also implicates innate immunity. A 13-AA gliadin peptide (LGQQQPFPPQQPY) induced IFN-γ, tumor necrosis factor-alpha (TNF-α), and interleukin 15 (IL-15) secretion from intestinal epithelial cells, macrophages, and dendritic cells (DCs) (35, 36). IL-15 is recognized as the dominant driver in refractory patients with CeD who exhibit villous atrophy and lymphoma without recent ingestion of gluten proteins (37, 38). IL-15 induces MHC class I chain-related (MIC) expression on enterocytes and the natural killer group 2D (NKG2D) expression on the intraepithelial lymphocytes. The T cell receptor-independent MIC-NKG2D interaction leads to enterocyte apoptosis and destructive lesions (35, 39–41).

Collective evidence illustrates that the immunogenicity of gluten proteins is complicated and thus safety evaluation of novel proteins for the CeD risk is challenging. The immune responses of novel proteins and products could be evaluated in patients with CeD using oral challenges. However, such an approach is costly and would involve risks for patients. The assessment of CeD-specific T cell reactivity can be performed using T cells derived from *in vitro* cultured intestinal biopsies or from the blood of patients with CeD after oral challenges. Yet those tests would require relevant clinical infrastructure and CeD subjects following informed consent. Animal models are available alternatives but require complicated evaluation and validation. Although there are commercial enzyme-linked immunosorbent assay (ELISA) kits that are widely employed to test for the presence of gluten proteins in various food products, the available antibodies (e.g., R5 and G12) may not target all proteins or peptides that pose a risk to CeD subjects. Furthermore, those ELISA tests do not predict peptides that might be deamidated by the endogenous enzyme in the lamina propria. Since many studies have reported that the gluten peptides are confirmed to elicit CeD reactivity, our goal was to establish a searchable database of the causative peptides as well as to develop simple but effective bioinformatics assessment tools for evaluating the potential risk of new dietary proteins for those with CeD. This publication describes the literature review used to identify the causative epitopes and proteins and describes the evaluation process used to validate the database, computer search routines, and risk assessment criteria. The practical utility of the database and the bioinformatics tools is then discussed.

## Materials and Methods

### Literature Review and Collection of CeD-Associated Peptides

The PubMed literature database (http://www.ncbi.nlm.nih.gov/pubmed) was searched for relevant publications using the keywords “celiac” and “coeliac” to identify studies investigating proteins and peptides capable of eliciting CeD pathogenesis. Native and deamidated peptides with documented evidence of stimulating CD4+ T cells restricted to MHC class II molecule DQ2.5, DQ2.2, DQ8, or DQ9 that lead to T cell proliferation with greater than a 2-fold stimulatory index or release of IFN-γ were collected. In addition to the immunogenic peptides, those with rigorous evidence of eliciting toxic reactions in the intestines of patients with CeD were collected as toxic peptides. Published toxic properties reported included one or more of the following indications: reduction in epithelial brush border alkaline phosphatase activity; increased intestinal permeability; reduction in enterocyte surface cell height (ECH) or reduction in villus height to crypt depth ratio (VH:CD); expression of epithelial apoptotic mediator ligand HLA-E molecule; maturation and migration of macrophage, DC, and CD4+ T cells to the lamina propria; or expression of inflammatory cytokines IFN-γ, TNF-α, and IL-15 (35, 36, 42–50). The peptides obtained from the selected publications were reviewed by five peer review panel members of the AllergenOnline.org database to confirm the selection rationale prior to constructing the database.

### Construction of the Database (Version 1, 2012) and the Bioinformatics Searching and Comparison Tools

The collected CeD peptides from literature searches were compared with the non-redundant National Center for Biotechnology Information (NCBI) protein database using the Basic Local Alignment Search Tool (BLAST) to identify identical peptides and the source proteins. Default BLASTP search parameters were used with an Expect threshold (*E*-score) of 10, matrix selection of BLOSUM62, gap costs of 11 for existence, and 1 for extension. The BLAST results showed that 425 native peptides from the 1,016 identified peptides had identity matches with 147 prolamins and glutelins of the Pooideae grass subfamily. The 147 proteins were then evaluated using the European Molecular Biology Laboratory-European Bioinformatics Institute (EMBL-EBI) multiple sequence alignment program ClustalW2. Identical sequences were removed and 68 non-redundant sequences were collected as representatives for CeD-associated proteins. Bread wheat (*Triticum aestivum*) and barley (*Hordeum vulgare*) are the major sources of the CeD reactive prolamins, which account for about 63% (43 out of 68 proteins) and 16% (11 out of 68), respectively (Table 1).

**Table 1:** Statistics of the AllergenOnline.org CeD peptide and protein database version construction and inclusion characteristics.

		**Version 1** **(released in 2012)**	**Version 2** **(released in 2018)**
References	Number of publication references^*^	68	**72**
	Publication year of references	1984–2012	1984–**2017**
Peptides	Number of peptides	1,016	**1,013**
	Number of native peptides	464	**465**
	Number of deamidated peptides	552	**548**
	Number of immunogenic peptides	998	**1,004**
	Number of CD4+ T cell reactive peptides	997	**1,003**
	Number of CD8+ T cell reactive peptides	1	1
	Number of toxic peptides (without T cell reactivity)	18	**9**
	Length of peptides (AA)	8–55	**9**−55
	Averaged length of peptides (AA)	16 ± 4	16 ± 4
Proteins	Number of proteins	68	**72**
	Number of proteins in *Triticum aestivum*	43	43
	Number of synthetic constructs in *Triticum aestivum*	1	1
	Number of proteins in *Triticum monococcum*	2	2
	Number of proteins in *Hordeum vulgare*	11	**12**
	Number of proteins in *Secale cereale*	6	6
	Number of proteins in *Avena sativa*	3	**6**
	Number of proteins in *Avena nuda*	2	2
	Length of proteins (AA)	20–800	20–800

*Changes between the two database versions are in bold font*.

* *Complete list of the 72 reference publications with the PubMed links is available on the database*.

The 1,016 identified CeD peptides and the 68 representative CeD proteins with the NCBI protein accession numbers were loaded into a MySQL relational database management system. The peptides with CeD-associated evidence and publication links were available in the browse function of the database. Query sequences entered by database users in the search window can be compared with CeD peptides by the exact identity match tool. The 68 representative CeD source proteins can be viewed in the browse function, and the sequences of query proteins can be compared for identity scores to each of the 68 representative CeD proteins by the full-length FASTA3 sequence alignment tool, version 35.04 (51). The peptide and protein database sections with complete references from 68 publications and the two sequence comparison tools were available for public use at http://www.allergenonline.org/celiachome.shtml from January 2012 until November 2017.

### Update of the Database (Version 2, 2018)

In 2017, an additional literature review was conducted and affirmed by six AllergenOnline.org peer review panel members. A total of 34 previously included peptides were removed from the database as they were less than nine AA long and were considered too short to effectively bind MHC and activate T cells. The core nine-AA peptides listed in the 2017 European Food Safety Authority (EFSA) guidance on allergenicity assessment of genetically modified plants and their predicted deamidated forms were added to the database (52, 53). Four additional publications were added to the database as references. One barley and three oat prolamins were identified through BLASTP and ClustalW2 bringing the representative proteins in the database to 72. The updated database version 2 was made public in October 2017 with a full explanation in January 2018 (Supplementary Tables 1, 3).

### Testing the Database to Define Criteria for Potential Risks for Eliciting CeD

Testing of version 1 of the database was conducted in 2012 and version 2 in 2018. Proteins tested included prolamins and glutelins from known CeD-associated species (e.g., wheat, barley, rye, and oat) and homologous proteins from sources outside of Pooideae that have a history of safe use for subjects with CeD (e.g., maize, sorghum, coix (adlay), millet, rice, and teff). The analyses were conducted using query sequences identified from the NCBI protein database from Pooideae sources and non-Pooideae sources using keywords: gluten, glutelin, glutenin, prolamin, prolamine, gliadin, hordein, secalin, avenin, zein, kafirin, coixin, canein, and pennisetin. In addition, each of the representative CeD protein sequences was searched against the non-redundant NCBI protein database by BLASTP using the Expect threshold of 10 and with the exclusion of the Pooideae proteins (NCBI taxonomic identifier: 147368), but excluding patented proteins. The 2012 results were compiled and sorted into four groups: (1) 2,666 prolamins from the Pooideae subfamily that may be considered possibly unsafe for patients with CeD; (2) 1,059 prolamins and prolamin related proteins from the grass subfamilies of Chloridoideae, Ehrhartoideae, and Panicoideae, sources that are considered to be safe for individuals with CeD; (3) 1,050 prolamin-like proteins from the dicotyledon class that are considered to be safe for patients with CeD; and (4) 48 unrelated proteins, obtained from the BLAST search from sources considered safe for patients with CeD (Table 2). Each sequence of the four groups was manually tested against the CeD database using both the exact peptide match and FASTA3 tools and the results (exact match hits and FASTA sequence homology scores [percent identity score, alignment overlap length, and *E*-score]) were recorded. The evaluation of the FASTA3 alignment scores was used to set the criteria for minimum percent identity and maximum *E*-scores that suggest risks of CeD. In 2018, similar NCBI searches were conducted, and version 2 database was tested using (1) 5,786 prolamins from the Pooideae subfamily; (2) 1,755 prolamins and prolamin related proteins from the grass subfamilies of Chloridoideae, Ehrhartoideae, and Panicoideae; and (3) 4,724 prolamin-like proteins from the dicotyledon class (Table 3). The results were used to validate the previously proposed criteria.

**Table 2:** FASTA sequence identity scores and alignments of the representative prolamin-like protein groups clustered by source organism types that were tested with the AllergenOnline.org CeD database version 1.

**Group**		**Number of proteins searched from NCBI**	**Contain exact CeD-associated peptides**	**Three best FASTA identity score results**		
				**Alignment overlap length (Length of the representative CeD protein)**	**% Identity to the CeD protein**	***E-*** **score**
I	Prolamins in Pooideae with CeD peptides	2,104^*^	Yes	827 (827)	100	2.8e−179
				287 (290)	100	7.8eb−81
				842 (838)	98.1	1.4e−195
	Prolamins in Pooideae without CeD peptides	562^*^	No	20 (20)	95	2.9e−05
				187 (288)	98.4	2.7e−45
				290 (288)	79.3	3.5e−63
II	Prolamins and prolamin-like proteins in Chloridoideae, Ehrhartoideae, and Panicoideae	1,059^*^^‡^	No	54 (52)	40.7	6.7
				12 (20)	66.7	1.9
				268 (360)	41	3.5e−17
III	Prolamin-like proteins in Dicotyledons	1,050^*^	No	68 (68)	33.8	2.3
				10 (20)	60	8.8
				121 (648)	30.6	1.8e−06
IV	Unrelated proteins (animals, fungi and microbes)	48^Δ^	No	29 (29)	58.6	3.8
				11 (20)	72.7	5.8e−03
				437 (439)	41.2	8.7e−25

* *Proteins were identified from the NCBI protein database using keywords: gluten, glutelin, glutenin, prolamin, prolamine, gliadin, hordein, secalin, avenin, zein, kafirin, coixin, canein, and pennisetin*.

‡ *35 proteins were obtained by BLAST, which searched the 68 representative celiac proteins against the NCBI Protein-Protein (non-redundant sequences) database with the exclusion of Pooideae (taxid: 147368) that had close to 45% identity over 100 AA and an E-score close to 1e-14. None had a direct CeD peptide match*.

Δ *proteins were obtained by BLAST searches with the 68 representative celiac proteins against the NCBI Protein-Protein (non-redundant sequences) database with the exclusion of Pooideae (taxid: 147368)*.

**Table 3:** Repeat of the FASTA sequence identity scores and alignments of the larger representative prolamin-like protein groups clustered by source organism types that were tested with the AllergenOnline.org CeD database version 2.

**Group**		**Number of proteins searched from NCBI**	**Contain exact CeD-associated peptides**	**Three best FASTA identity score results**		
				**Alignment overlap length (Length of the representative CeD protein)**	**% Identity to the CeD protein**	***E-*** **score**
I	Prolamins in Pooideae with CeD peptides	4,623^*^	Yes	828 (828)	100	1.0e−177
				439 (290)	100	1.6e−165
				455 (455)	100	8.4e−153
	Prolamins in Pooideae without CeD peptides	1,163^*^	No	291 (288)	98.6	3.7e−09
				264 (279)	98.9	1.1e−73
				266 (269)	98.5	3.6e−68
II	Prolamins and prolamin-like proteins in non-Pooideae monocots	1,755^*^^‡^	No	292 (250)	37.3	3.6e−09
				168 (181)	40.5	9.1e−09
				222 (222)	37.4	2.4e−08
III	Prolamin-like proteins in Dicotyledons	4,724^*^	No	305 (838)	32.1	1.6e−04
				372 (439)	28.8	9.5e−04
				253 (290)	29.2	9.3e−03

* * Proteins were identified from the NCBI protein database using keywords: gluten, glutelin, glutenin, prolamin, prolamine, gliadin, hordein, secalin, avenin, zein, kafirin, coixin, canein, and pennisetin*.

‡ *44 proteins were obtained by BLAST, which searched the 72 representative celiac proteins against the NCBI Protein-Protein (non-redundant sequences) database with the exclusion of Pooideae (taxid: 147368), that are close to significance based on percent identity and E-scores close to or below 1e-14. None contained a CeD peptide match, however one had 45–50% identity to four wheat glutens*.

### Database Tests Using Hypothetical Alanine-Substituted Alpha-Gliadin

Evaluation of the utility of the FASTA3 algorithm was tested using a sequence of α-gliadin of *Triticum aestivum* (NCBI accession number: CAB76964). This protein is one of the 72 representative proteins in the database and it contains 53 overlapping CeD-associated peptides identified with the exact sequence match tool. The sequence was altered *in silico* by substitutions in amino acid sequence to eliminate 53 exact peptide matches by amino acid substitutions of 13 alanine (A) residues in place of 12 Q and one tyrosine (Y) residues. For the second *in silico* modification, 11 theoretical substitutions were made with the addition of A in place of three serine (S), two glycine (G), four lysine (L), and one P and one Q amino acid residues. The two *in silico* modified alpha-gliadin sequences were evaluated using both the exact peptide match and FASTA3 tools. The exact match tool did not identify any possible risks, but the results with FASTA3 allowed us to consider criteria for meaningful alignments.

## Results

### The CeD-Associated Peptide and Protein Database

In reviewing publications of CeD-associated peptides, broad differences were noted in specificity, sensitivity, and severity of reactions (34). For example, pure oat products that were not contaminated by wheat, barley, or rye, were reported to be well-tolerated by the majority of the CeD consumers (54, 55). Oats has been considered to be safe for CeD consumers by the United States Food and Drug Administration (US FDA). However, avenin-reactive T cells that mediate the intestinal inflammation typical of CeD were identified in several patients with CeD (56–59), and the adequacy of a number of the oat safety evaluation studies remains controversial (60, 61). Since our goal is to include all known prolamin and glutelin peptides with scientific evidence of CeD induction to ensure that all CeD individuals are protected by our bioinformatics tools, the reported T-cell reactive avenin peptides were identified as risky for some CeD consumers, and 47 oat-derived peptides were included in the database.

Statistics of the database versions 1 and 2 are summarized in Table 1. Overall, 68 relevant publications published between November 1984 and October 2012 were selected to collect 1,016 overlapping prolamin and glutelin peptides 8–55 AA long. Most peptides included in the database are immunogenic. From the 1,016 collected peptides, 997 are CD4+ T cell reactive while only one is CD8+ T cell reactive. This highlights the role of T cell-mediated inflammation in CeD pathogenesis. In addition, more than half of the prolamin and glutelin peptides are post-translationally deamidated. Of the 997 CD4+ T cell reactive peptides, 445 were in native sequences and 552 were in predicted deamidated sequences.

We note that the core nine-AA peptide of the collected T cell epitopes is different for HLA-DQ2 versus HLA-DQ8, and that the identity of immunogenic peptides relevant in HLA-DQ8 is less frequently noted than those of DQ2 epitopes. Possibly some immunogenic peptides may not have been reported yet, and this database should be updated periodically to ensure accuracy. From the 1,016 collected peptides, 18 elicited pathological effects to the intestine without evidence of specific T cell activation. Most of the collected toxic peptides appeared to trigger innate immune responses, yet some of their sequences overlap the immunogenic peptides. Version 2 of the database comprises a total of 1,013 causative peptides and 72 representative CeD-associated proteins (Table 1).

### The Bioinformatics Tools for CeD Risk Assessment

**Figure 1:**
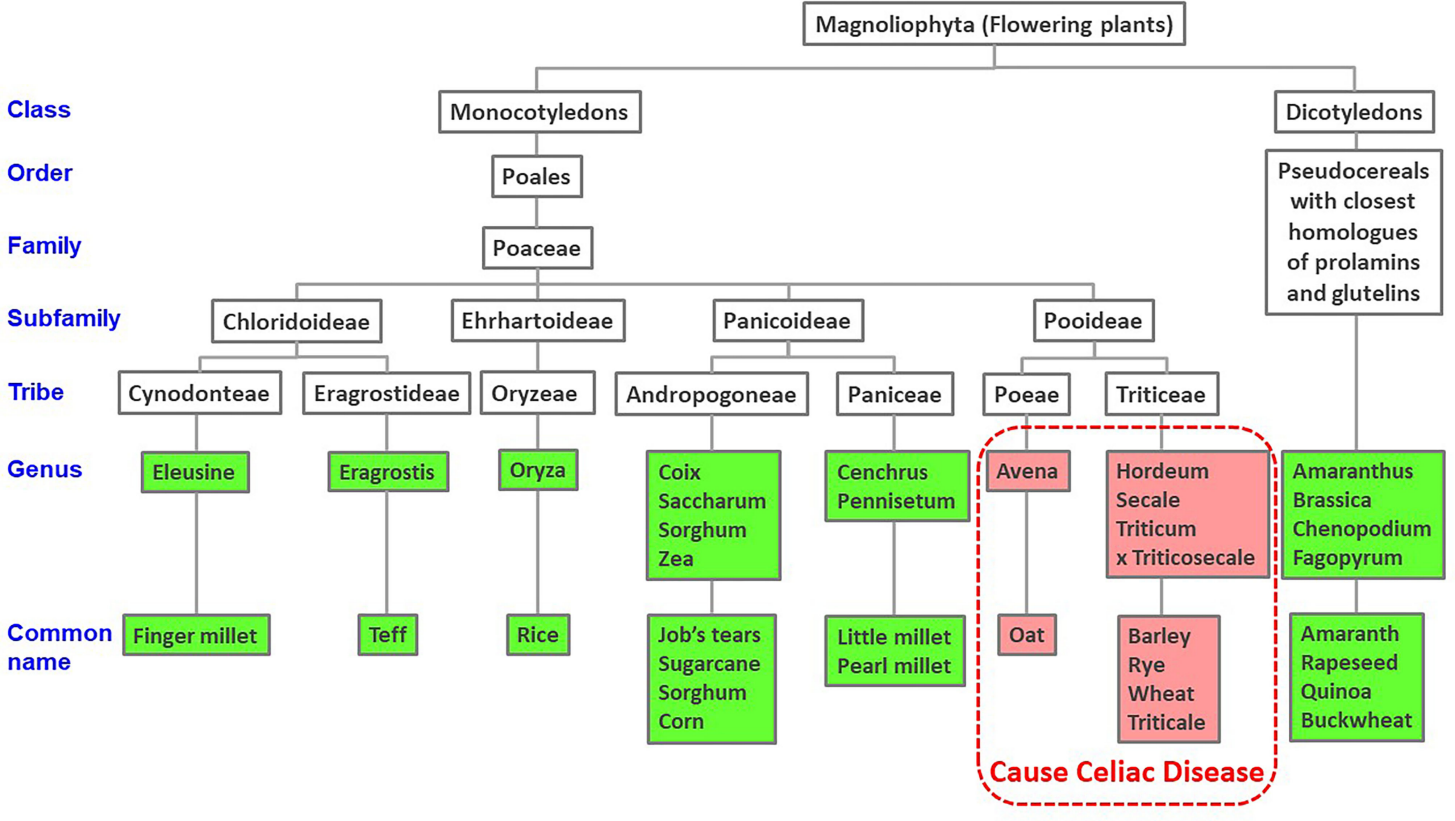
Taxonomic tree of cereal and dicotyledonous plants based on NCBI taxonomy. Published evidence of CeD safe foods show reactions only to grains of the Pooideae subfamily of grasses.

BLAST searches indicated that all the CeD-associated peptides are found only in the prolamin and glutelin storage proteins of the Pooideae subfamily or in predicted deamidation products of those sequences. There were no other cereals outside of Pooideae that are likely to elicit CeD including corn, rice, sorghum, or millets (Figure 1). Our recommendation for the database users is that any query sequences found to contain even one of the known 1,013 peptides could be a risk for some individuals susceptible to CeD. Those proteins should be tested further to evaluate risks with MHC II-restricted CD4+ T cells from subjects with CeD or relevant toxicity before being introduced into a gluten-free food. We recognized that approximately 20% (562 of 2,666 for the first FASTA analysis and 1,163 of 5,786 for the second FASTA analysis) of the gluten-like proteins identified from Pooideae do not contain any of the known CeD reactive peptides (Table 2). Those proteins might be safe for CeD consumers; however since some T-cell reactive or toxic peptides may remain undiscovered, testing would be a conservative choice (62). Therefore, we recommend using the full-length FASTA sequence alignment tool to identify query sequences that may lack an exact peptide match to the 1,013 peptides, but may include previously undefined CeD reactive peptides to confer an added layer of confidence.

**Figure 2:**
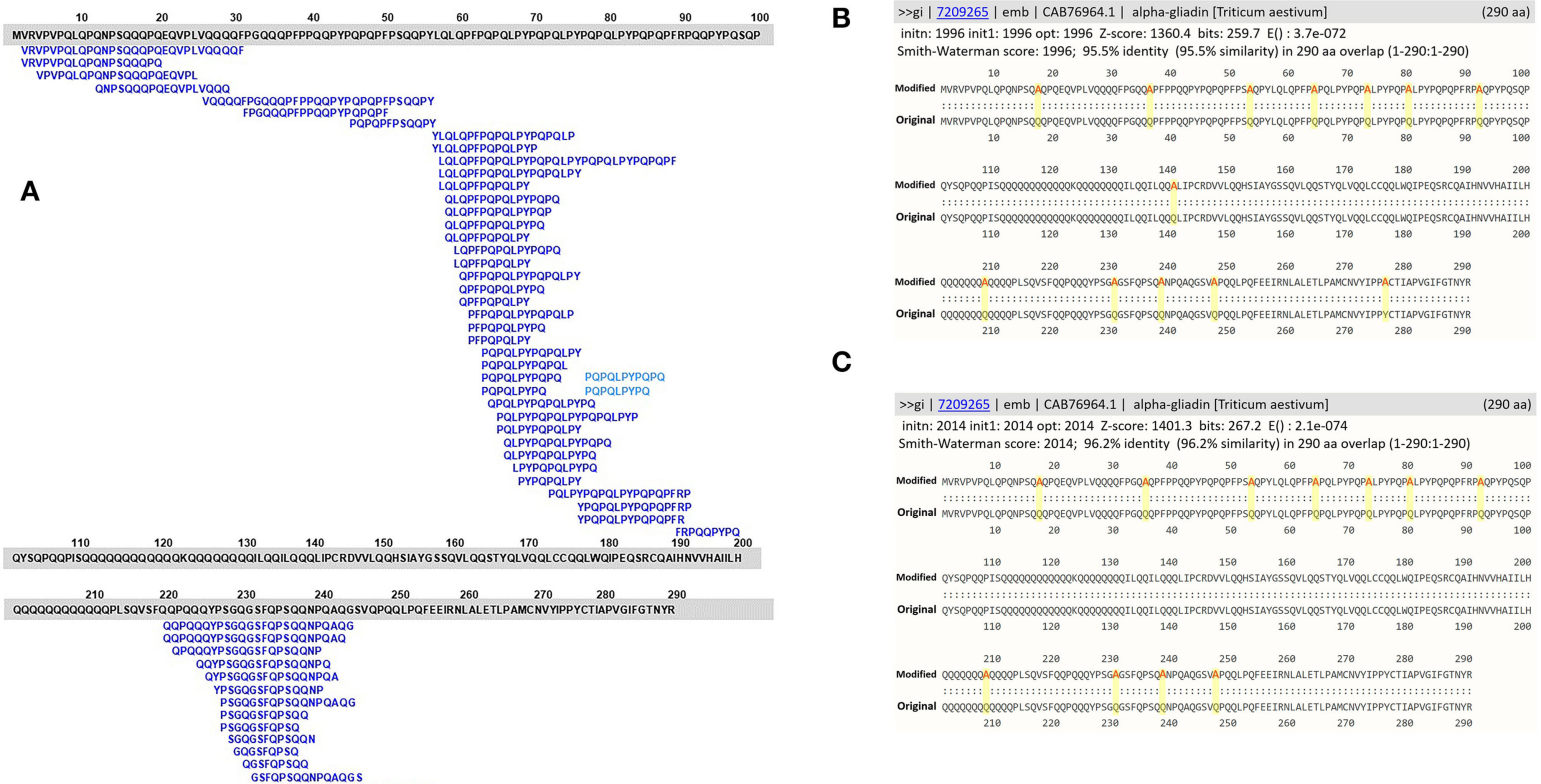
**(A)** Amino acid sequence alignments of an α-gliadin (NCBI accession number: CAB76964) with 53 overlapping CeD-associated peptides identified with the exact sequence match tool; **(B)** full FASTA sequence alignment results with homology scores of the α-gliadin theoretically substituted with 13 alanine residues; **(C)** full FASTA sequence alignment results with homology scores of the α-gliadin theoretically substituted with 11 alanine residues.

To reinforce the utility of the FASTA alignment tool, *in silico* amino acid substitution in positions of exact CeD peptides of a CeD-associated α-gliadin (NCBI accession number: CAB76964) was conducted. The substitutions were made so that each of the known 53 overlapping CeD-associated peptides were no longer native and they are not identical to CeD peptides (Figures 2A–C). When the modified sequences were searched with the full FASTA3 sequence alignment tool, they showed > 95.5% identity full-length alignment to the original α-gliadin with *E-*scores smaller than 1.1e-78. These conservative substituted sequences might still be recognizable by the DQ2 or 8 restricted T-cells of patients with CeD. Without laboratory or clinical evidence of safety, it is prudent to flag these two sequences as probable risk factors for CeD. It is clear when using the full FASTA3 sequence alignment that careful evaluation of matching data is required since the query sequence can align with segments of the 72 representative CeD protein sequences in regions that do not harbor antigenic CeD determinants (Figure 2A, AA 98–219 and 255–290).

There are many gluten-like proteins in other grass subfamilies outside of Pooideae and some in dicotyledonous plants that have a clear history of safe consumption by those with CeD. A large number of these protein sequences were collected and used for testing with the first FASTA analysis in 2012 to provide identity scores, alignment overlap lengths, and *E*-scores that were used to set limits to differentiate conservative safety guidelines that are useful to identify possibly risky sequences (Table 2). The alignment results indicated that the 562 Pooideae prolamin sequences lacking any exact match to the known CeD-associated peptides could have high identity FASTA alignments up to 98.4% over half-sequence length (187/288) and with an *E*-score of 2.7e-45 and can be up to 79.3% identical for a full-length (290/288) alignment with an *E*-score of 3.5e-63 to the representative CeD proteins. In contrast, a number of query sequences in non-Pooideae grass subfamilies (group II) were found to align over their full length with representative CeD proteins, and none were more than 43% identical with the representative CeD proteins (Table 2). Nearly all the query sequences in group II represent very short alignments with the representative CeD proteins and with the minimum *E*-score of 3.5e-17. In addition, full-length alignment comparison analyses of the prolamin-like sequences from dicotyledon class (group III) resulted in even lower identity scores and larger *E*-score values, while short overlaps (10/20) had up to 60% identities with *E*-scores as large as 8.8 (Table 2). Finally, 48 protein sequences from animals, fungi, or bacteria (group IV) were compared with the database by full-length alignment, and the FASTA results indicated that these 48 proteins could produce full-length (437/439) alignments with up to 41.2% identity and with the smallest *E*-score of 8.7e-25. The maximum identity was 72.7% over the half-sequence lengths (11/20) alignment, having a minimum *E*-score of 5.8e-03 (Table 2). The second FASTA analysis was performed in 2018 using version 2 of the database, and a total of 12,265 identified sequences led to the consistent results with those obtained from the first FASTA analysis (Table 3). In that analysis, of the 1,163 Pooideae prolamins lacking the exact peptide match (group I), the best FASTA alignment can be up to 98.9% identical over a full-length (264/279) alignment with an *E*-score of 1.4e-73 to the representative CeD proteins. Of the 1,755 query sequences from non-Pooideae grass subfamilies (group II) and 4,724 query sequences from dicotyledons class (group III), the best is a full-length FASTA alignment (168/181) of 40.5% identical with an *E*-score of 9.1e-09 to the representative CeD proteins.

In summary, the exact sequence match searches and the full FASTA sequence alignment searches indicated that the tested proteins in groups II, III, and IV from both the 2012 and 2018 analyses, are unlikely to implicate any of these proteins in CeD pathogenesis. The proteins found to contain no known CeD-associated peptides and when searched with FASTA comparison with the representative CeD proteins do not have significant identity matches and have relatively large *E*-scores. The percent identity describes the proportion of amino acids that are identical in alignment taking into consideration sequence and spacing. The *E*-score is a parameter describing the expected number of hits when searching a database of a particular size based on a log scale. Small numbers (e.g., *E* = 1e-20 or less) indicate likely significant matches.

## Discussions and Conclusion

Several approaches can be undertaken to evaluate the CeD-associated immune response to novel foods including *in vitro* T cell studies using lines and clones and T cells obtained from patients after oral challenges and *in vivo* schemes using specific animal models. However, our database and algorithms aim at providing simple yet effective screening tools with low rates of false positive and false negative results. Identity to even one of the known CeD-associated peptides indicates a potential risk, and thus our proposed exact peptide sequence match tool is the most definitive comparison. Among the existing gluten-associated databases, the AllergenOnline.org CeD database contains the largest number of identified CeD-associated sequences (63, 64), and this allows higher quality and more versatile comparison. Since it has been a while since 2018, an expedited update was conducted in April 2022 to include newly identified CeD-associated peptides (65) and subsequently it will be given a rigorous evaluation as described for the 2018 version. Six more formal nine-AA CeD-relevant T cell epitopes, 22 CD4+ T cell reactive peptides, and four representative protein sequences were included bringing a total of 1,041 peptides and 76 protein sequences in our database. The full FASTA sequence alignment algorithm appears to be a useful tool to identify proteins with possible CeD risks while recognizing that the knowledge of the CeD-associated peptides is incomplete. The sequence comparison provides an additional assessment and compensates for the lack of identification of all CeD-associated peptides or cases of mutation that might remove exact match sequences, but possibly not diminish the CeD reactivity.

Upon evaluating the 4,823 (first analysis) and the 12,265 (second analysis) sequences of proteins with known risks of CeD and homologs from outside of Pooideae considered to be safe for CeD consumers, we compared alignment scores and characteristics of the two sequence groups. When compared with the representative CeD-associated proteins, all sequences from the latter group fall into one of the following alignment characteristics: having <45% sequence identity; having an *E*-score >1e-14; and aligned with many gaps or aligned with less than a full 100-AA overlap. These did not align with regions harboring the antigenic determinants. Although a single bioinformatics threshold cannot be attained, these four major observations together signify a less likely risk of CeD and are useful for careful evaluation of the FASTA alignment result. As a result, any query sequences identified with the FASTA comparison outside these alignment characteristics should not be defined as CeD safe without further verification. In fact, higher percent identity, lower *E*-scores, or longer the full alignments suggest a higher probability of sequence identity to the known CeD-associated proteins and thus appear to be of potential risk for eliciting CeD that must be critically evaluated further for the safe use for individuals with CeD.

**Figure 3:**
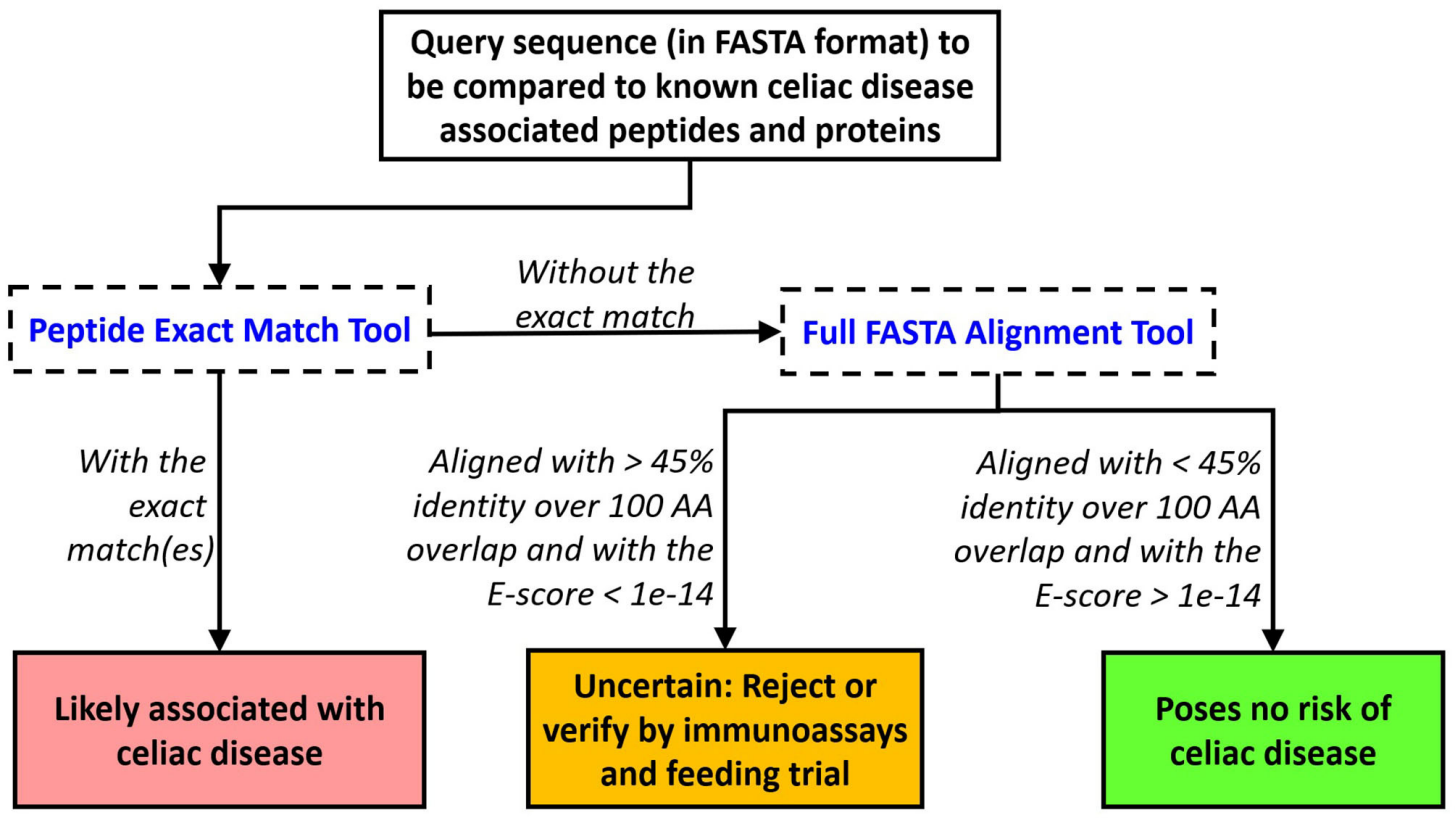
Proposed evaluation criteria to predict the likelihood of a query protein to cause elicitation of CeD. An exact match to any of the 1,041 peptides indicates probable rejection. Alternatively, a FASTA3 alignment with an *E*-score limit of 1e-14 and minimum alignment length > 100 AA with an identity percent of the protein at 45% should trigger testing or rejection.

Our bioinformatics scheme and evaluation criteria for assessing novel food proteins for eliciting CeD are depicted in Figure 3. First, each query sequence should be screened for the presence of any of the 1,041 CeD-associated peptides using the peptide exact match tool. An exact match to any of the known CeD-associated peptides indicates a probable risk. Query sequences without matches to the known CeD-associated peptides are then evaluated to identity high-scoring matches to the 76 representative CeD proteins using the full FASTA alignment tool. Sequence alignments to any of the representative CeD proteins with at least 45% identity and an *E*-score <1e-14 over 100 AA alignment suggest that they may harbor antigenic determinants. The significance of matches by either method can be verified by testing using the appropriate specific T cell (66).

In 2017, the EFSA Panel on Genetically Modified Organisms (GMO) published guidance on the allergenicity assessment of genetically modified plants stating that any new protein expressed in a GMO must be evaluated for safety to CeD consumers. The guideline was referred to our CeD database but also suggested sequence evaluation with the 4 AA (Q/E-X1-P-X2) motifs (52). Song et al. (67) however demonstrated that the Q-X1-P-X2 motif searches for potential CeD risk yielded poor selectivity and suggested including our FASTA comparison evaluation to improve the risk assessment efficiency (67).

New varieties of crops are being developed as new food sources. Perennial grain crops offer potentially sustainable new food sources. A wheatgrass *Thinopyrum intermedium*, also in the Pooideae subfamily, has been under development for 30 years as a possible alternative to annual wheat (*Triticum* sp.) (68). Our preliminary analysis however indicated that 31 predicted probable proteins from the *T. intermedium* genome contain multiple known CeD-associated peptides. It is highly likely that consumption of *T. intermedium* grain would also trigger CeD in some individuals. The predicted *T. intermedium* proteins also match more than 30 known wheat allergen sequences in the AllergenOnline database version 21, suggesting a risk for consumers with a wheat allergy.

In summary, our CeD peptide and protein database with bioinformatics tools have been robustly evaluated by us and by other users in the past 10 years and proven to offer an effective screening system for the identification and analysis of CeD-associated peptides and proteins for a thorough food safety evaluation (67, 69–77). The curated database and tools are available for public use free of charge at http://www.allergenonline.org/celiachome.shtml, and an update and validation of the risk evaluation criteria will be continued by our peer review panel members.

## Data Availability Statement

The datasets presented in this study can be found in online repositories. The names of the repository/repositories and accession number(s) can be found in the article/Supplementary Material.

## Author Contributions

PA, ST, and RG: conceptualization and writing—review and editing. PA, JW, and RG: methodology. JW: software. PA, AT, BB, FF, ST, and RG: validation. PA and MA: formal analysis, investigation, and writing—original draft preparation. PA and RG: resources, data curation, and visualization. RG: supervision and project administration. ST and RG: funding acquisition. All authors contributed to the article and approved the submitted version.

## Funding

This research was funded by the Food Allergy Research and Resource Program (FARRP), University of Nebraska. Limited funding was provided by Unilever SEAC and by Nuseed NA and earlier by six individual biotechnology industrial sponsors of the www.AllergenOnline.org database from 2009 to 2012. PA received a Royal Thai Government Scholarship for his Ph.D. studies. MA received a Government of Egypt fund for his Ph.D. studies.

## Conflict of Interest

RG declares limited funding from six biotechnology companies from 2009 to 2012 for support of the database management. Unilever SEAC and NuSeed Americas provided limited funding to the AllergenOnline.org database from 2018 to 2021. These companies did not contribute to or see the article prior to submission. The remaining authors declare that the research was conducted in the absence of any commercial or financial relationships that could be construed as a potential conflict of interest.

## Publisher's Note

All claims expressed in this article are solely those of the authors and do not necessarily represent those of their affiliated organizations, or those of the publisher, the editors and the reviewers. Any product that may be evaluated in this article, or claim that may be made by its manufacturer, is not guaranteed or endorsed by the publisher.
